# Protein Manipulation via Dielectrophoresis: Theoretical Principles and Emerging Microfluidic Platforms

**DOI:** 10.3390/mi16050531

**Published:** 2025-04-29

**Authors:** Zuriel Da En Shee, Ervina Efzan Mhd Noor, Mirza Farrukh Baig

**Affiliations:** Centre for Manufacturing and Environmental Sustainability (CMES), Faculty of Engineering and Technology, Multimedia University, Ayer Keroh, Malacca 75450, Malaysia; zurielshee@gmail.com (Z.D.E.S.); farrukhbaig@mmu.edu.my (M.F.B.)

**Keywords:** microfluidic, dielectrophoresis, microelectrode, lab-on-a-chip, proteins

## Abstract

Dielectrophoresis (DEP) has been widely employed in microfluidic platforms for particle or cell manipulation in biomedical science applications due to its accurate, fast, label-free, and low-cost diagnostic technique. However, the application of the DEP technique towards protein manipulation has yet to be extensively explored due to the challenges of the complexity of protein itself, such as its complex morphologies, extremely minuscule particle size, inherent electrical properties, and temperature sensitivity, which make it relatively more challenging. Furthermore, given that protein DEP investigation requires entering the micro- to nano-scale level of DEP configuration, various challenging factors such as electrohydrodynamic effects, electrolysis, joule heating, and electrothermal force that emerge will make it more difficult in realizing protein DEP investigation. This review study has discussed the fundamental theory of DEP and considerations toward protein DEP manipulation. In particular, it focused on the DEP theoretical principle towards protein, protein DEP application challenges, microfluidic platform considerations, medium considerations, and a critically reviewed list of protein bioparticles that have been investigated were all highlighted.

## 1. Introduction

Dielectrophoresis has been proven as a versatile manipulation technique commonly used in a microfluidic platform to transport, separate, accumulate, and characterize micro- to nano-size bioparticles in a microfluidic system [1]. Its versatile capabilities have given numerous advantages for clinical application and laboratory diagnosis in current modern research.

“Dielectrophoresis”, with its analog “DEP”, was coined by Herbert A. Pohl in around 1950, where it is derived from *phorein*, the Greek word meaning an effect where a particle is ‘carried’ due to the result of its dielectric properties. In scientific terms, it is an electrostatic phenomenal which describes the induced motion in a suspended particle by its interaction with an applied non-uniform electric field. Until today, numerous modern scientific applications benefit from this discovery [2,3,4,5].

The DEP technique is commonly used to manipulate dielectric particles in a non-uniform electric field by the induction of DEP force (F_DEP_) [6]. DEP is commonly used as a technique in microfluidic for particle or cell manipulation. It uses the principles of polarization and the motion of bioparticles in applied electric fields. This technique has been regarded as a promising technique in biomedical science applications due to its label-free, accurate, fast, low-cost diagnostic technique [5]. DEP can influence both conducting and non-conducting (dielectric) particles, with the necessary electric fields generated using either direct current (DC) or alternating current (AC) sources; however, AC fields are more commonly utilized in microfluidic and biological applications due to their superior control over heating and electrolysis effects [7]. Fundamentally, all particles—regardless of their material composition—exhibit some degree of dielectrophoretic behavior when exposed to a non-uniform electric field [8]. This phenomenon typically occurs when a particle is suspended in a fluid medium and subjected to an electric field, generally within a frequency range of 10 kHz to 100 MHz [9]. Under these conditions, the electric charges within both the particle and the surrounding medium redistribute, resulting in a net force that causes the particle to move toward regions of either higher or lower electric field intensity, depending on its dielectric properties relative to the medium [8].

The application of DEP in protein analysis has demonstrated significant potential in clinical diagnostics due to its label-free operation, rapid processing time, and high sensitivity. DEP has been particularly effective in the detection of disease-specific protein biomarkers across various medical conditions [10]. For instance, in prostate cancer diagnostics, DEP-based platforms have been used to target biomarkers such as prostate-specific antigen (PSA), human glandular kallikrein 2 (hK2), Annexin A3 (ANXA3), beta-2-microglobulin (β2M), microseminoprotein-beta (MSMB), serum amyloid A (SAA), and Engrailed-2 (EN2). Recent studies suggest that the multiplexed detection of these biomarkers provides improved diagnostic accuracy compared to single-marker approaches [11]. Similarly, in breast cancer, DEP aids in the identification of critical protein biomarkers including estrogen receptor (ER), progesterone receptor (PR), human epidermal growth factor receptor 2 (HER2), and cancer antigen 27.29 (CA27.29) [12]. These biomarkers play an essential role in early diagnosis and the development of personalized treatment strategies, although challenges related to expression variability and specificity persist. Overall, the integration of DEP with microfluidic and biosensing technologies continues to advance its relevance and practicality in clinical settings.

**Figure 1:**
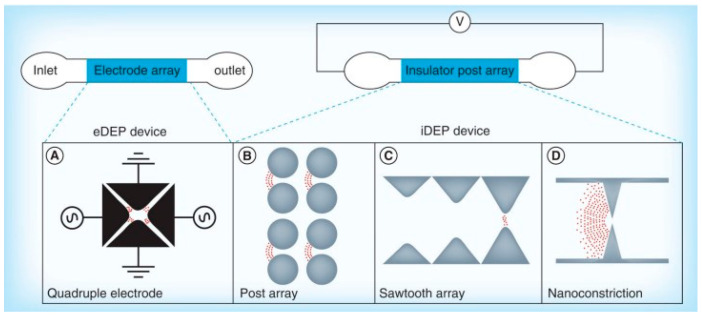
Conceptual classification of microfluidic dielectrophoresis (DEP) platforms [13].

Since the initial exploration of DEP for protein manipulation, researchers have developed a wide range of microfluidic devices employing both electrode-based and insulator-based configurations. These platforms have demonstrated significant success in enabling the controlled movement and separation of bioparticles at the microscale. Figure 1 presents an overview of the most widely used DEP architectures in protein-related studies [13]. DEP systems generally fall into two main categories based on how they generate the required non-uniform electric field: electrode-based DEP (eDEP), which relies on microfabricated electrodes to create field gradients, and insulator-based DEP (iDEP), which utilizes strategically shaped insulating structures within the microchannel to distort a uniform electric field and induce DEP forces.

## 2. DEP Theoretical Background

DEP is an electrokinetic phenomenon which can be induced on a dielectric particle that is suspended in a liquid medium. When the particle is exposed to a non-uniform electrical field, it will be polarized and the electrical charges on the particle are induced to make up a dipole [5]. Due to the particle’s dipole interaction and the spatial gradient of the electric field, each half of the dipole experiences unequal Columbic forces, resulting in a net force imposed on the particle that leads to a motion on the particle. This is known as the DEP force (F_DEP_). This DEP force can push the particle either towards or off from the strong electric field region depending on the polarizability between the particle and the medium. The movement of the particle towards a high electric field region is called positive dielectrophoresis response (pDEP) and the movement of the particle towards a low electric field region is called negative dielectrophoresis response (nDEP).

The equation of a time-averaged DEP force exerted on a spherical particle is expressed as below [1]:

(1) $$<F>=2\pi r^{3}\varepsilon_{m}\varepsilon_{0}Re\left[f_{CM}\right]\nabla E_{RMS}^{2}+4\pi r^{3}\varepsilon_{m}\varepsilon_{0}Im\left[f_{CM}\right]\sum_{x,y,z}E_{RMS}^{2}\nabla \varphi$$

The above Equation (1) is the time-averaged DEP force which consists of two major independent terms. The first term of the equation is proportional to the real part of the Clausius–Mossotti factor $Re\left[f_{CM}\right]$, from which $f_{CM}$ is a complex variable that is dependent on the spatial non-uniformity of the electric field [1]. Often, the first term is commonly known as ‘classical DEP force’. Depending on the $Re\left[f_{CM}\right]$ polarity, the force applied on the particle can either be pushed towards or repealed from the strong electric field region.

On the other hand, the second term is proportional to the imaginary part of the $Im\left[f_{CM}\right]$ and is associated with the spatial non-uniformity of the phase component. The second term is known as the ‘travelling wave DEP force’ (F_TW-DEP_). This force is applied to the particle in the direction of the wave propagation or away from it, depending on the $Im\left[f_{CM}\right]$.

In Equation (1), ε_m_ is denoted as the absolute relative permittivity of suspension medium, while ε_0_ = 8.854 × 10^−12^ F/m is the permittivity of the vacuum. The spherical particle’s radius is denoted as r (or rₑₓₜ, where rₑₓₜ refers to the particle’s external or effective radius used in modeling DEP interactions. E_RMS_ is the root-mean-square of the applied electric field in AC, and $Re\left[f_{CM}\right]$ is the real part of Clausius–Mossotti (CM) factor, which is further defined in Equation (4), and $Im\left[f_{CM}\right]$ is the imaginary part of the Clausius–Mossotti (CM) factor. Lastly, φ represents the phase component of the electric field.

For the case of a homogeneous spherical particle in the suspension medium, the general time-averaged DEP force amplitude can be expressed by the classical DEP force as Equation (2) [6].

(2) $$F_{DEP}=2\pi \varepsilon_{m}\varepsilon_{0}r_{ext}^{3}Re\left[{ƒ}_{CM}\right]\nabla E_{RMS}^{2}$$

**Figure 2:**
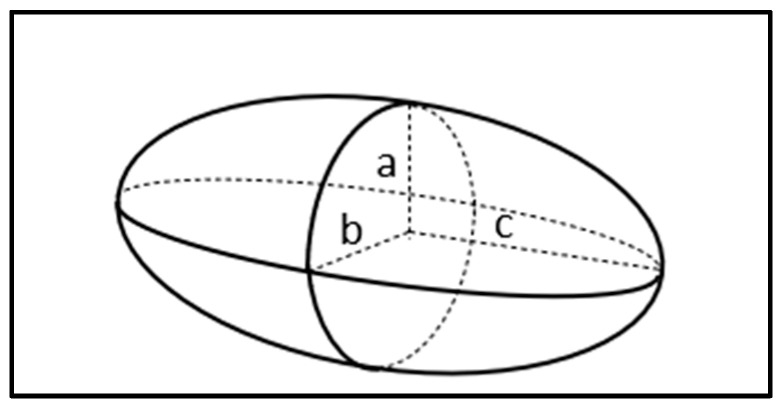
Ellipsoid geometry figure.

For an ellipsoid shape or rod-shaped particles, which are depicted in Figure 2, the three axes’ radii are given by a, b, and c (b > a = c), and the volume is defined by $v=\frac{4}{3}\pi abc$. Therefore, the time dependent DEP force equation is given by the following [9,10]:

(3) $$F_{DEP}=\frac{2\pi abc}{3}\varepsilon_{m}Re\left[{ƒ}_{CM}\right]\nabla E^{2}$$

The equation of the real part of Clausius–Mossotti factor $Re\left[f_{CM}\right]$ is given in Equation (4).

(4) $$Re[{ƒ}_{CM}]=\frac{\varepsilon_{p}^{*}-\varepsilon_{m}^{*}}{\varepsilon_{p}^{*}+2\varepsilon_{m}^{*}}$$

where the subscripts “*p*” and “*m*” refer to the particle and medium, respectively. $\varepsilon_{p}^{*}$ and $\varepsilon_{m}^{*}$ are the complex permittivity with respect to particle and medium which can be defined as in Equation (5).

(5) $$\varepsilon_{p}^{*}=\varepsilon_{p}\varepsilon_{0}-j\frac{\sigma_{p}}{\omega },\;\;\;\varepsilon_{m}^{*}=\varepsilon_{m}\varepsilon_{0}-j\frac{\sigma_{m}}{\omega }$$

in which σ_p_ and σ_m_ is the electric conductivity of a particle and medium, respectively. ω is the angular frequency of the applied electric field, defined as ω = πƒ, where ƒ is the frequency of the electric field and $j=\sqrt{-1}$.

The DEP response caused by the DEP force on the particle can either be pushed towards or away from the regions of high electric field and is dependent on the polarity of the CM factor (ƒ_CM_). In the case where ƒ_CM_ < 0, the particle is said to be pushed towards the strong electric field region; this motion response is called positive DEP (pDEP). Conversely, for the case of ƒ_CM_ < 0, the particle is repelled from the high electric field regions to the low electric field region, and this response is a negative DEP (nDEP) [1].

If the ƒ_CM_ > 0, the particle will be pushed towards the high electric field regions. As such, the motion response is termed as positive DEP (pDEP). Conversely, if ƒ_CM_ < 0, the particle is repelled from the strong electric field regions to the weak electric field region. This response is a negative DEP (nDEP) [1]. For ƒ_CM_ = 0, the particle will not move due to the net DEP force becoming zero. The specific frequency at this moment is known as the crossover frequency or zero force frequency. This phenomenon occurs when the real part of the effective polarizability of the particle and the suspending medium are equal to each other [5].

**Figure 3:**
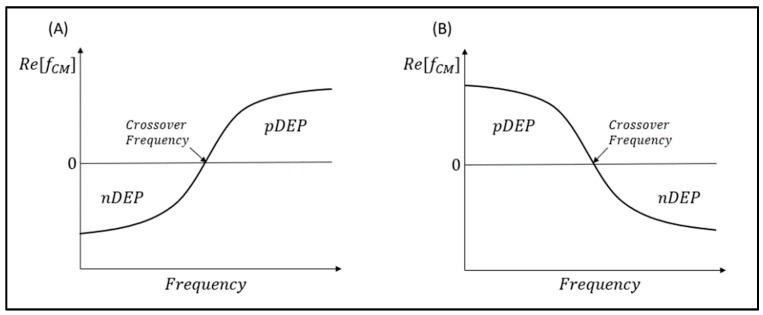
The DEP spectrum of Re[ƒ_CM_] vs. frequency of a polarizable particle. (**A**) When σ_p_ < σ_m_ or ε_p_ > ε_m_; (**B**) when σ_p_ > σ_m_ or ε_p_ < ε_m_.

We must understand that the CM factor is established by two main variables, which are the conductivity and permittivity. The conductivity is responsible for controlling low frequency DEP activity, while the permittivity is responsible for controlling high frequency DEP behavior [5,11]. Hence, the relationship between applied frequency and the real part of CM factor Re[ƒ_CM_] can generally be classified into two cases as shown in Table 1 below. The corresponding DEP response across the frequency spectrum is illustrated in Figure 3.

## 3. External Forces in DEP System

In a microsystem consisting of an aqueous medium and protein particle mixture, the motion or forces experienced by the particle are usually much more complex, with various factors and forces that directly affect the manipulation of the particles. The external forces that arise in the DEP manipulation include drag force, AC electro-osmotic force, electrothermal forces, Brownian motion, and buoyancy force. To achieve success of DEP protein manipulation, the DEP force must be large enough to overcome these external forces. Table 2 has summarized the generation of the external forces and the solution to circumvent them.

## 4. DEP Temperature Effects

When applying an electric field to the DEP system, temperature may rise due to joule heating ($\propto \sigma E^{2}$). As the current flows through a fluid medium, the fluid is heated due to the rapid movement of electrons, and it can be expressed as the following [15]:

(6) $$∆T\sim L^{2}{\left|E\right|}^{2}$$

The length that characterizes the electric field variations is denoted by L, and E is denoted the electric field strength. It has been suggested that reducing the DEP device scale can help decrease the temperature rise.

The thermal gradient across the fluid medium will result in convective circulation. Moreover, the dielectric properties of the fluid medium are temperature dependent. The medium conductivity gradient is influenced by the electrical field which may contribute to inducing unwanted fluid flow. As the temperature rises from the result of a high electric field, it will inevitably impact protein physiology to an extent that it causes protein denaturation and aggregation formation. The temperature gradient can be expressed as below [22]:

(7) $$∆T\approx \;\sigma V^{2}L^{3}$$

L is the length scale of the system; V denotes the voltage, and the conductivity is denoted as σ. The change in temperature will be more apparent at a larger scale of applied voltage, since the change in temperature is proportional to the applied voltage.

In an AC electric field, the increase in AC frequency will lead to the heating of polarizable dielectric particles, which is known as dielectric heating. This phenomenon appears when the dipole in the dielectric particle constantly reorients in a high frequency AC electric field to adjust with polarity change; therefore, heat is dissipated, owing to the molecular friction [23]. The temperature rise, *Δϑ*, in the dielectric particle due to the generated heat is given by the following:

(8) $$∆\vartheta =\frac{\omega {\varepsilon^{\prime \prime }}_{m}\varepsilon_{0}E^{2}V}{\alpha A_{c}}\left(1-e^{-\left(\frac{\alpha A_{c}}{mc}\right)t}\right)$$

*ε″_m_* is the imaginary part of complex permittivity, $\varepsilon_{0}$ is the permittivity of free space, E is the electric field, *V* is the volume of suspending medium, *α* is heat transfer coefficient, *A_c_* denoted as effective surface for heat convection, ω is the angular frequency, *m* the mass, *c* the specific heat capacity, and *t* is the elapsed time.

## 5. Protein DEP Theoretical Considerations

Proteins play an important role in the human body to provide various essential functions in regulating it, such as an antibody as the defense mechanism to viruses and bacteria. Proteins can act as an enzyme to carry out chemical reactions, while some proteins act as messengers to transmit signals to coordinate biological processes. Proteins also provide structure and support for cells and as transport or storage for small molecules [24]. Undoubtedly, proteins are essential in the human body due to them encompassing most aspects of body function and regulation. However, this is a nascent field, as there are limited studies that have performed the DEP technique on protein application [5], and a substantial theoretical model describing protein DEP is still lacking [25]. 

Proteins are huge, complex molecules made up by the building blocks known as amino acids. The amino acids are attached in a long polymer chain by peptide bonds and fold into complicated 3D structures. The number of amino acids in a protein is dependent on the size of the protein, which can be calculated by its total molecular mass and with a unit of daltons (Da) [26]. The folded proteins are often viewed as approximate and with a spherical geometry. The equivalent radius for the protein can be based on their hydrodynamic (Stokes) radius which is given in Equation (9) [1,27,28].

(9) $$r_{hydrodynamic}=\frac{k_{B}T}{6\pi \mu D}$$

where k_B_ is the Boltzmann constant in J/K, D represent the diffusion coefficient in m^2^/s, and T is the temperature in unit K.

The theoretical framework for protein frequency-dependent DEP and its dipolar response is extremely complex due to proteins itself being a form of polypeptides that are built up by various amino acids which result in forming a tertiary structure. Protein dipoles are formed largely by their permanent dipoles which associate with the molecular composition of proteins, which typically have a few hundred Debye. Other contributing factors include: (a) the spatial arrangements of polarizable groups by the bonds in the polypeptide backbone, (b) the amino acid side chains polar and charged groups or even α-helices from specific motifs in protein, (c) protein dipole moment associated with solvent and solvent-protein interactions, and (d) electrical double layer (EDL) ions distribution polarization which also contribute to the characteristics of frequency-dependent DEP [25].

Therefore, the classical DEP force equation approach in Equation (2) would be oversimplified and does not provide substantial information to the complex structure of protein details [25]. By further extending the classical DEP Equation (2) with the assumption of a globular structure protein as an ellipsoid shape, the dielectrophoretic response can be formulated in such in Equation (10) [29].

(10) $$F_{DEP}=\frac{4\pi abc}{3}\varepsilon_{m}\left(\frac{\sigma_{p}-\sigma_{m}}{Z\sigma_{P}+(1-Z)\sigma_{m}}\right)\nabla E^{2}$$

where the ellipsoid’s semi principal axes are denoted as a, b, and c (a > b = c). The term in the bracket is known as the polarization factor denoted by P, or some called as the modified real part of the Clausius–Mossotti (CM) factor Re(ƒ_MCM_) as shown in Equation (11) [30]. Z is called the depolarization factor as shown in Equation (12).

(11) $$P=\left(\frac{\sigma_{p}-\sigma_{m}}{Z\sigma_{P}+(1-Z)\sigma_{m}}\right)$$

(12) $$Z=\frac{bc}{2a^{2}e^{3}}\varepsilon_{m}\left[ln\left(\frac{1+e}{1-e}\right)-2e\right]$$

e is denoted as the eccentricity, which is given by in Equation (13):

(13) $$e=\sqrt{1-\left(\frac{bc}{a^{2}}\right)}$$

## 6. DEP Applications in Clinical Diagnostics

The application of DEP in protein analysis has demonstrated significant potential in clinical diagnostics due to its label-free operation, rapid processing, and high sensitivity. DEP has been particularly effective in the detection of disease-specific protein biomarkers across various medical conditions [31]. For instance, in prostate cancer diagnostics, DEP-based platforms have been used to target biomarkers such as prostate-specific antigen (PSA), human glandular kallikrein 2 (hK2), Annexin A3 (ANXA3), beta-2-microglobulin (β2M), microseminoprotein-beta (MSMB), serum amyloid A (SAA), and Engrailed-2 (EN2). Recent studies suggest that the multiplexed detection of these biomarkers provides improved diagnostic accuracy compared to single-marker approaches [32]. Similarly, in breast cancer, DEP aids in the identification of critical protein biomarkers including estrogen receptor (ER), progesterone receptor (PR), human epidermal growth factor receptor 2 (HER2), and cancer antigen 27.29 (CA27.29) [33]. These biomarkers play an essential role in early diagnosis and the development of personalized treatment strategies, although challenges related to expression variability and specificity persist. Overall, the integration of DEP with microfluidic and biosensing technologies continues to advance its relevance and practicality in clinical settings.

**Figure 4:**
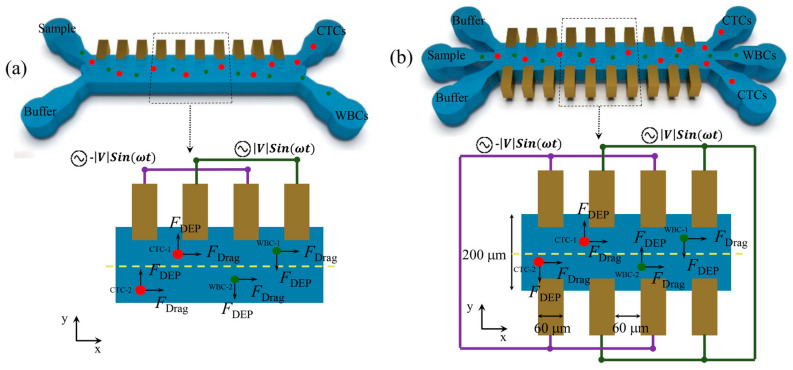
(**a**) Conventional DEP-based systems. (**b**) PDMS-based microfluidic device with 3D sidewall electrodes [34].

A DEP-based microfluidic system was developed for the selective separation of cancer cells while preserving their viability [34]. This system introduces a significant innovation by maintaining cell integrity during the separation process—an area where conventional methods often fall short. Ensuring cell viability is critical for reliable downstream analysis and accurate cancer diagnostics. Notably, the system incorporates double-sided 3D sidewall electrodes, which significantly enhance electric field distribution compared to traditional planar electrode designs. As illustrated in Figure 4, the comparison between planar and 3D sidewall electrodes in a PDMS-based microfluidic channel demonstrates the improved field uniformity and strength achieved through this advanced electrode configuration, enabling more efficient and gentle cell manipulation.

**Figure 5:**
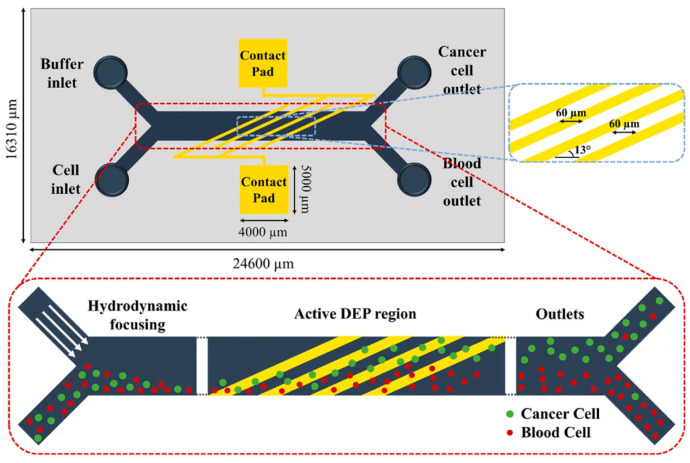
DEP microfluidic chip designed to isolate MCF7 breast cancer cells [35].

DEP-based microfluidic systems have emerged as a powerful platform for the detection and separation of circulating tumor cells (CTCs) [35]. The integration of DEP into microfluidic devices offers a transformative, label-free approach to CTC capture and analysis by leveraging the intrinsic dielectric properties of cells. Unlike conventional methods that rely on surface markers, DEP enables the selective manipulation of CTCs while preserving their viability and structural integrity—essential for downstream applications such as genetic profiling, drug sensitivity testing, and cell culturing. Figure 5 illustrates a DEP microfluidic chip with a magnified view of the electrode and microchannel layout, highlighting the device’s functional architecture. The advancement of DEP-based microfluidic technology holds significant promise for enhancing cancer diagnosis, real-time monitoring, and personalized treatment strategies. Ongoing innovation in this area is expected to yield more sensitive, efficient, and accessible tools for early cancer detection and therapeutic decision-making.

## 7. Fabrication Technologies for DEP Microfluidic Platforms

The development of DEP microfluidic platforms is intrinsically tied to the evolution of microfabrication technologies. Traditional fabrication methods, including soft lithography with PDMS, photolithography, and wet or dry etching, have long served as the foundation for early DEP device construction. However, the recent rise of additive manufacturing has significantly broadened the design and fabrication landscape, enabling rapid prototyping, high-precision structuring, and the integration of complex three-dimensional architectures.

Additive manufacturing techniques such as digital light processing (DLP) [36] and stereolithography [37] allow for high-resolution, layer-by-layer fabrication of microfluidic channels, and electrode features with remarkable speed and flexibility. These methods offer substantial benefits, including enhanced design freedom, reduced material waste, and the seamless incorporation of functional components such as embedded electrodes, optical windows, or valve mechanisms [38,39].

Emerging studies have underscored the transformative potential of 3D printing in the context of DEP device fabrication. A novel digital manufacturing method was introduced for constructing monolithic capillaric circuits (CCs) with embedded microconduits, demonstrating that fully functional microfluidic systems can be fabricated using accessible DLP 3D printers [40]. In a related effort, researchers presented a 3D-printed microfluidic device for automated, pressure-driven microchip electrophoresis, optimized for detecting preterm birth (PTB) biomarkers. This device achieved sensitive peptide separation under refined operational conditions, illustrating its value for early risk assessment in clinical diagnostics [41]. Additionally, the “print-pause-print” protocol was developed and enabled stereolithographic fabrication of transparent, multimaterial, biomicrofluidic chips, facilitating precise material alignment for advanced applications in biomolecular analysis, organ-on-a-chip platforms, and tissue engineering [42]. More recently, a nanoporous, gas-permeable polyethylene glycol diacrylate (PEGDA) ink designed for 3D printing organ-on-a-chip (OoC) devices was introduced. This novel material overcomes limitations of PDMS by supporting higher oxygen permeability, improved cell adhesion, and complex geometry formation, thereby enhancing cell viability in long-term culture [43].

Together, these innovations represent a paradigm shift in DEP microdevice fabrication, offering a more accessible, scalable, and customizable alternative to conventional methods. As additive manufacturing technologies continue to mature, their integration with DEP systems is poised to accelerate the development of next-generation, microfluidic platforms for a wide range of biomedical, diagnostic, and therapeutic applications.

## 8. Case Studies and Performance Metrics of DEP-Based Microfluidic Systems

Recent developments in DEP-based microfluidic systems have shown promising experimental outcomes for practical biological and clinical applications. Here, we summarize selected studies in Table 3 that report key performance metrics to guide researchers in system development and optimization.

## 9. DEP Experimental Application of Protein Manipulation

### 9.1. Trapping

The immobilization of proteins to a region of interest in the DEP device is called the trapping technique, which is achieved by the inducement of specific electric field to the protein. To attain this phenomena, the DEP force must overcome various other forces in the system such as hydrodynamic force, thermal force, electrokinetic force, diffusion and friction force, and Brownian motion [25]. Typically, the suitable electrode geometry for this application that have been investigated are the quadruple electrodes, nanopipette, circular post array, sawtooth, and nano-constrictions.

### 9.2. Focusing

In contrast to trapping technique to immobilize the protein, this technique allows us to focus the proteins to stream towards a narrow or confined stream. This technique has been demonstrated to be successful in separation or sorting targeted particles. The DEP force has to overcome the Brownian diffusion and electro-osmosis force to be able to focus the particle. Triangular post array and planar electrodes geometry have been employed in DEP focusing application.

### 9.3. Separations

DEP force is greatly dependent on polarizability; therefore, by adopting the characteristic of different polarizability of the particles in the mixture, we can achieve separation of the particle through the dieletrophoretic responses, such as pDEP and nDEP. This technique has potential in protein application due to the advantage of such frequency-dependent characteristic enabling the separation process to be ‘tunable’. By controlling the applied electric field with specific a magnitude of frequency to render the polarization, we attain the desired DEP response. Interdigitated electrodes devices have been demonstrated in protein DEP separation; however, the separation is still highly influenced by the particle size and polarizability in such chromatography devices.

## 10. Protein DEP Microfluidic Platform

In general, there are two types of DEP devices that are commonly utilized in the investigation of protein DEP. These are the electrode-based DEP devices (eDEP) and insulator-based DEP devices (iDEP) [25]. The eDEP device is the most commonly adopted in protein DEP investigation. DEP was performed on the microfluidic device which embedded metallic electrodes within the sample channel, which allows the current flow to create an electric field gradient [44]. iDEP is the device that incorporates insulator structures within the microfluidic device. The electrodes are placed at the opposite ends of the device and an array of insulator posts are located in between the electrodes to create a non-uniform electric field [48].

### 10.1. Electrode-Based DEP (eDEP)

The advancement in microfabrication techniques that enable the fabrication of electrodes into smaller dimension sizes and desire patterns aided in the realization of the electrode-based DEP system. The electrode-based DEP was performed on microfluidic devices, utilizing a pair of non-uniform geometry pattern of microelectrodes embedded within the channel in direct contact with the analytes. This arrangement enables the generation of a non-uniform electric field from the irregular shape of electrodes.

The eDEP technique is the most commonly seen technique that is employed in the field of protein DEP due to its frequency-dependent component advantage. eDEP has the advantage to generate greater electric field gradients with minimum applied voltage; however, these high electric fields only surface at the vicinity of the electrodes [25] and have an inherent limitation such as electrode fouling [49], spatial limitations, and may increase sample temperature due to electrodes having direct contact interaction with analytes.

#### 10.1.1. Metal Electrode Limitation in eDEP

In DEP settings, high electric fields are often required to trap proteins. eDEP is able to achieve high electric field gradients with a low applied voltage. However, several challenges may arise when it is used with protein samples. At first, due to the complex electrochemical reactions, significant electrolysis may occur at the microelectrodes. As a result, the occurrence of gas bubble generation will interfere the protein trapping process or even damage the trapped proteins and degrade the electrodes [50]. Furthermore, protein denaturing may happen due to proteins being prone to being attracted to the microelectrode’s metal surface. Conjointly, microelectrode functionality may decrease significantly due to fouling arising from protein sample interaction [49]. Lastly, it may not be compatible as the low ionic strength of the sample solution will lead to the unfolding of proteins.

#### 10.1.2. Electrode Contamination in eDEP

Electrode contamination remains a persistent challenge in eDEP systems, particularly due to electrochemical reactions at the electrode–electrolyte interface during prolonged operation or high-voltage application. Such contamination can alter electric field distribution, reduce device performance, and degrade protein samples.

Recent studies have proposed several solutions to this issue. One common approach involves coating the electrode surface with a thin dielectric passivation layer such as silicon dioxide (SiO₂) or hafnium oxide (HfO₂), which can effectively reduce Faradaic reactions while maintaining electric field penetration [51,52,53]. Hybrid electrode materials, such as gold–PDMS composites or graphene-integrated electrodes, have also shown improved chemical stability and reduced fouling [54,55]. Moreover, the use of optimized AC waveforms (e.g., bipolar or pulsed AC) has been reported to minimize the accumulation of electrolysis products [56,57,58]. Additionally, continuous buffer flow or periodic flushing of the microchannel can prevent buildup on the electrodes [59]. These strategies collectively contribute to maintaining the longevity and functionality of eDEP platforms during sensitive protein manipulation.

### 10.2. Insulator-Based DEP (iDEP)

Insulator-based DEP (iDEP) is a relatively new technique which build upon on the principle of spatially non-uniform electric field components. A non-uniform electric field is generated by incorporating an array of insulator constrictions or posts embedded in the microfluidic device and a pair of external electrodes, each placed on opposite ends (inlet and outlet) of the microfluidic device in direct contact with the medium [60]. In this arrangement, the applied DC uniform field will move around the insulating structures, thereby creating an electric field gradient. The electric field and voltage can be controlled by changing the size and geometry of the insulating posts [48]. iDEP provides a relatively easy fabrication process and due to its multiple array posts arrangement enabling the non-uniform field to cover the entire depth of microchannel. The electrodes in iDEP are less prone to fouling due to the electrodes being in minimum contact with analytes, thereby minimizing electrode reaction. Only a few examples of iDEP being used on proteins have been reported. Nonetheless, the benefit it provided has demonstrated to be promising.

## 11. Protein DEP Platform in AC and DC

DEP can be operated in either an alternating current (AC) or direct current (DC) that induces a non-uniform electric field gradient, in which a dielectrophoretic force is exerted on the polarized particle in the suspension medium that induced motion. This non-uniform electric field gradient can be created by (a) using a non-uniform electrode geometry in an AC field or by (b) embedding insulating obstacles of non-uniform geometry in a uniform DC field [22,61]. Based on these two methods, two types of DEP system devices, which have mainly been employed in the application of protein DEP investigation, have been developed, which are the electrode-based DEP (eDEP) and insulator-based DEP (iDEP). More detail of these reported devices has been tabulated in the following section.

### 11.1. Direct Current of Protein DEP

When using a DC setting for DEP, a significant amount of voltage is required to achieve a strong DEP force. As a consequence, the temperature in the channel will rise due to the joule heating effect [15,17]. An alternative evasive method that is being used to resolve this is using weak buffer solutions or nonaqueous solutions as the suspending medium. Moreover, a finned structure or rectangular channels in the microfluidic platform also show efficacy in reducing the joule heating effect [62]. In order for the particle to experience higher DEP force in DC-DEP, Adrienne R. Minerick has suggested that the non-uniformities in the electric field must exist over length scales comparable to the particle (<10 mm) of interest [63].

#### Protein Exhibits Less Conductivity in DC Electric Field

Protein particles behave less conductively when under DC electric fields. This has been reported by Lapizco-Encinas et al. in their work of examining the DEP response of the protein with the condition of zero CM factor settings [49]. In order to achieve a CM factor equal to zero, Lapizco-Encinas et al. selected the BSA protein as the model organism, with a reported 25 µS/cm conductivity and accompanied by artificial suspended medium, which was also adjusted to a conductivity of 25 µS/cm to match each other to give a zero CM factor. Theoretically, the ideal DEP response to be expected is no particle motion. However, they observed a nDEP response. Through this, they investigated and suggested that it is due to protein particles behaving less conductively under DC electric fields, because when in the same 25 µS/cm conductivity medium, the BSA with less conductivity than 25 µS/cm BSA will be more prone to a nDEP response.

### 11.2. Alternative Current of Protein DEP

AC-DEP can be used to control virtually any form of particle in any type of medium [64]. The particle motion primarily results from DEP force. Therefore, based on Equation (2), the key influence in particle motion by DEP force was the electric field gradient and the particle volume, which was defined by its radius. In other words, small particles like proteins will require higher electric field gradients to compensate for their intrinsically miniscule radius. However, increasing the voltage to obtain a higher electric field gradient will have several side effects, such as water electrolysis, joule heating, or electrothermal effects [65]. To perform molecular DEP, it is inevitable that an electrode architecture capable of generating substantial strong electric field gradient with low voltage is required.

AC-DEP has the advantage of allowing high field strength while avoiding undesirable side effects such as water electrolysis, electro-osmosis currents, and electrophoresis of charged proteins [65]. Moreover, AC-DEP has the advantages of conveniently exploiting frequency-dependent effects, which can easily control the electric fields exerted on particles by adjusting parameters such as magnitude, frequency, wave shape, wave symmetry, and phase [64]. The advantages and disadvantages of DC-DEP and AC-DEP are summarized in Table 4.

## 12. Protein DEP Platform Design Consideration

In many DEP applications, a temperature effect due to joule heating is a common phenomenon. Indisputable joule heating is attributed to the failure of DEP trapping when the buffers used have high conductivities [66]. The consequences of high electric fields applied also results in a rise in temperature due to joule heating and electrolysis of the suspending medium. This constraint significantly restricts the effectiveness of the DEP force in manipulate protein particles.

In protein DEP, the trapping force strength dwindles to a minuscule amount in correlation to the common micro- to nano-size of protein bioparticles. Furthermore, the use of high conductivity mediums enhance the localized joule heating, thereby leading to the generation of an electrothermal flow that further dissipates the already low degree of trapping force [66,67]. Under these circumstances, every amount of DEP force is vital in contributing to the effectiveness of protein DEP manipulation. In the work of Chaurey et al., they investigated the influence of device scaling on electrodeless insulator dielectrophoresis (iDEP) with highly constricted channels of small channel depth to examine the balance of DEP force versus electrothermal force on streptavidin protein biomolecules with a reduced size (~5 nm) with medium conductivity in range (1–10 S/m). From the results, they proved that the reduction of depth of highly constricted channels to submicron levels will bring drastic reduction on joule heating, thereby allowing a greater threshold of voltage and medium conductivity to be applied in rapid DEP application [68]. On the other hand, reducing high voltages and electrodynamic effects to maintain DEP power is a feasible option too. Several studies also reported that the design of electrodes to a micro- to nano-size and with a smaller electrode gap size have shown efficacy in solving the problems caused by high voltages and electrohydrodynamic effects in the microfluidic system [27,69,70]. In the case of insulator-type DEP, a nano-scale geometry insulator design is recommended, as it will vastly improve DEP trapping by requiring less voltage to produce the same electric field as a micro-scale geometry insulator [71].

The two previous concerns, high electric field generation and joule heating, can be alleviated with the advancement of photolithography fabrication for micro to nanometer-sized electrodes. It has been shown that a narrow gap the size of the electrodes for eDEP, and a nanoscale gap between the insulators in iDEP, will drastically increase the electric field gradient [72]. Maria Laux et al. suggested the suitable thickness of electrode for protein DEP is in the range of 40 nm to 150 nm, which can help in increasing the field strength at the edge of electrodes, thereby enhancing the field gradient. A sharp curvature electrode pattern, compared to orthogonal pin electrodes, has also shown to provide a higher field strength [65].

## 13. Protein Particle Consideration and Factors

### 13.1. Protein Aggregation and Agglomeration

A protein’s polypeptide chain has a high intrinsic proclivity for self-assembling into a variety of misfolded aggregates. Protein denaturation, when near to the metal surface of electrodes due to high temperature effect from joule heating, is another factor that promotes aggregation formation [50]. Protein aggregation can come in different forms, ranging from dimers to closely ordered fibrils containing thousands of protein molecules [73]. Protein aggregation can be a problematic factor, especially in vitro studies of proteins in the case of DEP application. Several studies have indicated that the DEP response for protein aggregates and protein monomers are expected to be different [74,75,76]. It is undeniably a hassle that influences the DEP response.

To get rid of this nuisance, the works of Nakano et al. have demonstrated that by adding the zwitterionic detergent CHAPS to the buffer, we can prevent protein aggregation formation, as CHAPS has the ability to improve protein solubility without a noticeable change in buffer conductivity. From their experiment on iDEP streamlining of immunoglobulin G (IgG) and bovine serum albumin (BSA), it was reported that this buffer additive is effective in preventing the protein aggregate formation and can be used in bioanalytical applications [75,77]. Furthermore, the study by Hölzel et al. suggested that in order to exclude the aggregation, a DEP trap using fluorescence correlation spectroscopy to determine and clarify the presence of monomeric species of R-phycoerythrin from a single molecule has shown effectiveness [27].

Maintaining the medium pH at the physiological value helps in maintaining the protein’s structure. The work of Agastin et al. used a HEPES buffer, a zwitterionic sulfonic acid buffering agent that is commonly used in cell culture and is very effective in maintaining physiological pH. Three different proteins—BSA, fibrinogen, and lectin—were suspended in HEPES buffer with a pH near to the physiological value of 7.4. The buffer conductivity was reduced to 9.5 × 10^−4^ S m^−1^ by dilution with double distilled DI water to a concentration of 0.25 mM. It showed efficacy in maintaining the essential structure and stability of the proteins and DNA used in the experiments [78].

### 13.2. Protein Absorption

In microfluidic devices, PDMS channels are susceptible to nonspecific protein adsorption, with nonspecific adsorption of protein amyloid aggregates often found along the channel surface [79]. To avoid unwanted protein adsorption onto the PDMS surface, Nakano et al. used 500 µM tri-block copolymer F108 to coat the channel overnight prior to the experiment [30]. Additionally, Pluronic^®^ F108 was used as a surfactant in the medium by Nakano et al. to keep the cells from sticking to the surface of a microfluidic device. It is reported that the tri-block copolymer F108 forms micelles in the solution at a temperature at 25 °C and a concentration of 3 mM. Micelle formation affects the protein DEP response due to the reduced surface conductivity of the protein micelle. As a result, it changes the sign of the Clausius–Mossotti factor [77].

## 14. Medium Consideration for Protein DEP

### 14.1. Medium pH

Protein behavior in DEP is greatly influenced by pH, as it will greatly affect the net charge of the protein and cause aggregation, therefore influencing DEP application [80]. Some pH-dependent factors influencing dielectrophoresis are electro-osmosis, electrophoresis, and DEP mobility [77]. The influence of medium pH gradients in iDEP with Immunoglobulin G protein were studied both experimentally and numerically. A transition in pH was changed towards the acidic spectrum in the time scale of 10 min under constant buffer conductivity and applied voltage. It has been shown that a lower pH medium is able to suppress electro-osmotic flow in PDMS microchannels, thereby enhancing DEP force against electro-osmotic flow [76]. However, at pH levels below 6.5 and above 8, protein aggregation is favored to occur. This is ascribed to the degradation and conformation changes within protein [77]. Additionally, the same analysis on the protein concentration factors with varying pH from 8 to 6.5 revealed that protein concentration is enhanced at higher pH levels [77]. Thus, the reported suitable working range is suggested at a pH of 6–8 for IgG.

### 14.2. Medium Conductivity

Another factor is the usage of high-conductivity buffer solutions that may lead to undesired electrothermal effects or even cause excessive osmotic stress for biological analytes. Low suspension medium conductivity (below 100 mS/m) and low frequency (below 15 kHz) will cause electrode polarization to happen. Electrode polarization can occur at the interface between the medium and the electrode surface in a DEP device when the electrode is in direct contact with the medium. This is due to the discontinuity in charge carrier species between the metal and liquid suspension, as in metal, the current is carried by electrons while in fluid, it is carried by ions. This electrode polarization leads to an electric potential loss in the medium, therefore reducing the DEP force to the particle and eventually causing a reduction in particle manipulation capabilities. In addition, the electrode polarization will also result in AC electroconvection, which is the local heating around electrodes, and bubble formation at the electrode vicinity and eventually dissoluting [15].

Nonetheless, appropriate low conductivity of the suspending medium is a common consideration factor to prevent joule heating in DEP application. The influence of the buffer conductivity in relation to protein DEP behavior has been studied by Nakano et al. By adjusting the salt concentration, the buffer conductivity varied between 0.01 and 0.04 S/m as tested on iDEP with IgG. The intensity of the streamlined protein trapping was reported to decrease with an increase in buffer conductivity [75]. On the other hand, Liao et al. introduced the molecular damming effect to enhance ultrafast mass transport of protein enrichment in nanoscale electrodes and channels in DEP that is compatible to use with high-conductivity buffers [81]. Table 5 summarizes all the major protein DEP approaches that have been investigated in this protein DEP field.

## 15. Concluding Remarks

In this review article, a comprehensive review of the challenges and considerations in dielectrophoresis toward protein application was presented and critically reviewed. The DEP theoretical considerations for protein application have been discussed, and the critical aspects such as temperature factors, external forces, and other side effects that are often observed in protein DEP systems have been highlighted. The present state of DEP protein manipulations can be broadly classified as trapping, focusing, and separation. The contemporary DEP techniques and microfluidic platforms employed in protein dielectrophoresis, eDEP and iDEP, have been summarized by their characteristics and highlighted for their benefits and drawbacks. Protein DEP considerations and circumvention solutions were critically expounded in several major areas such as protein DEP, protein particles, microfluidic platform, and suspending medium. Dielectrophoresis is a novel protein manipulation technique that provides significant benefits in protein analysis and detection in research and clinical studies. Despite the fact that protein dielectrophoresis application is a complex procedure, understanding the relationships and factors in protein DEP will considerably ease and enhance the effectiveness of the protein dielectrophoresis research.

## Figures and Tables

**Table 1:** The relationship of polarity of Re[ƒ_CM_] DEP response.

Condition		Response	DEP Spectrum
Case 1	σ_p_ < σ_m_	Re[ƒ_CM_] is negative at low frequencies and positive at high frequencies.	Graph A
	ε_p_ > ε_m_		
Case 2	σ_p_ > σ_m_	Re[ƒ_CM_] is positive at low frequencies and negative at high frequencies.	Graph B
	ε_p_ < ε_m_		

**Table 2:** External forces and competing effects that affect the DEP response.

Forces	Generation	Solution	References
Drag Force	Induced by the interaction of particle with the medium flow field.	For sub-micrometer particles and small systems prefer at high frequencies.	[12,14]
Electrothermal Force	Caused by the joule heating or external heating from microscope lighting that affects the temperature gradient, altering the permittivity and conductivity gradient of the fluid and inducing an electrical body force.	Reduce high electrical field frequency and high voltage.	[15,16,17]
AC electro-osmotic Force	Caused by a nonlinear electrokinetic effect of induced-charge electro-osmotic flow around electrodes from applied AC voltage.	Prevent operation in a lower frequency range and small system size.	[15,18,19]
Buoyancy Force	Buoyant forces are proportional to the volume and do not scale favorably as the device size is reduced.	Buoyancy dominates at average system sizes of the order of greater than 1 mm.	[12,20]
Brownian Motion	The random movement of particles suspended in a fluid by the result of the molecules colliding or induced by thermal effects.	For a particle with size larger than 1 µm, Brownian motion is negligible.	[15,21]

**Table 3:** Case studies and performance metrics of DEP-based microfluidic systems.

Application Area	Study Focus	Performance Metrics	References
Circulating Tumor Cells (CTCs)	DEP-based microfluidic system with 3D sidewall electrodes for viable cancer cell separation.	High capture efficiency; 95% viability of MDA-MB-231 cells	[34]
Point-of-Care CRP Detection	Sequential flow-through microfluidic chip with DEP-enhanced immunoassay.	Detection limit: 47 pg/mL; linear range: 0.01 ng/mL–100 µg/mL.	[44]
Bacteria Separation from Blood	DEP microfluidic device for isolating *E. coli* and *S. aureus* from blood cells.	Electrode distance of 60 µm, an applied potential of 100 V, a frequency of 0.1 MHz, and a buffer-to-sample flow rate of 3.	[45]
Label-Free Protein Detection	Coupling of liquid chromatography with a quartz crystal microbalance (QCM) platform.	Sensitivity limit of 100 μg/mL for protein detection.	[46]
Cell Sorting for Leukemia Diagnosis	n-DEP platform for sorting leukemia cells (k-562) based on dielectric properties.	83% separation efficiency, cell viability by culture process is approximately 85%.	[47]

**Table 4:** Advantages and disadvantages between DC-DEP and AC-DEP.

	**DC-DEP**	**AC-DEP**
Electrode	Insulator-based electrodeless DEP	Planar electrodes Interdigitate electrodes (IDE) Quadrupole electrode 3D geometry electrode
Advantages	Ease and simplicity of use. Insulative and chemically inert microfabricated structures. Less prone to fouling. Removeable positioned electrodes.	Frequency dependent. Easy to control electric field. Reduce side effects of electro-osmotic currents, and electrophoresis. Reduce joule heating. Suppress electrochemical reactions on electrode surfaces.
Disadvantages	Electrolysis and DC electrophoresis occur at higher DC field strength. Joule heating at insulating structure. Greater dead volumes. Require concentrating and recovering samples after use.	Promote electrolysis of water. Microchips reusability reduced due to fouling. Inevitable electrical reactions.

**Table 5:** List of various proteins investigated by different DEP manipulation techniques, microelectrode platforms, and DEP configurations.

Target Protein	MW (kDa)	DEP Operation	DEP Platform Characteristics	Electrode Gap Spacing	Suspending Medium	Voltage	Frequency	Electric Field	Investigators	References
**eDEP**										
Avidin Concanavalin A Chymotrypsinogen A Ribonuclease A	68 52 25 13.7	Trapping and Separation	Interdigitated electrodes	4, 15, 55 µm	Distilled water	0–15 V	0.001–1 MHz	3 × 10^6^ V/m	Washizu et al., 1994	[74]
Avidin	68	Separation	Interdigitated electrodes	15 µm	Distilled water	0–40 V	1 kHz	3 × 10^6^ V/m	Washizu et al., 1994	[74]
Avidin	67–68	Trapping	Polynomial electrodes	2, 6 µm	KCL solution	10 V_P-P_	<9 MHz (pDEP) >9 MHz (nDEP)	-	Bakewell et al., 1998	[82]
Insulin BSA IgM	6 66 900	Separation	DEP chromatography corrugated shape electrodes	7 µm	PBS	–	1 Mhz	0.6 × 10^6^ V/m 1 × 10^6^ V/m 1.4 × 10^6^ V/m	Kawabata et al., 2001	[83]
Actin	42	Focusing	Quadrupole electrodes	7, 12 µm	M-buffer	10 V_P-P_	0.1–30 MHz (pDEP)	1.5 × 10^6^ V/m	Asokan et al., 2003	[84]
BSA	68	Trapping	Quadrupole electrodes	5, 10, 20 µm	Deionized water	10 V	0.05–5 MHz (pDEP) 2–3 kHz (optimum)	10^6^ V/m	Zheng et al., 2004	[85]
R-phycoerythrin	240	Trapping	Triangular planar electrodes	500 nm	RPE solution, Ultrapure water	10 V_rms_	0.1–5 Mhz 1 Mhz (optimum)	10^21^ V^2^/m^3^	Hölzel et al., 2005	[27]
Microtubules	-	Trapping	Indium–tin-oxide (ITO) electrode	100 ± 1 µm	Low-salt buffer solution	22 V_rms_	0.01–5 MHz	5 × 10^4^ V/m– 9 × 10^4^ V/m	Minoura et al., 2006	[86]
Albumin	66	Focusing and Trapping	Zipper-shaped electrodes	100 µm	Distilled water	10 V_P-P_	0.5–1.4 Mhz	-	Hübner et al., 2007	[87]
BSA	66.5	Trapping	3D nanopillar with a bevel of 30°	10 µm ⌀50 nm	Deionized water	–	1 MHz (pDEP)	3 × 10^6^ V/m	Yamamoto et al., 2007	[88]
Kinesin-microtubules	120	Focusing	Castellated electrodes	20 µm	BRB12 buffer	30, 35 V_P-P_	0.1–2.5 MHz (pDEP)	10^20^ V^2^/m^3^	Uppalapati et al., 2008	[89]
Amyloid peptide nanotubes	-	Trapping	Micro-patterned electrodes	1	Distilled water	1–10 V_P-P_	0.1–10 MHz (pDEP)	10^21^ V^2^/m^3^	Castillo et al., 2008	[90]
Streptavidin	60	Trapping	Carbon nanotube (CNT) tips	⌀5–20 nm	Pure water	2 V_P-P_	1 MHz	–	Maruyama et al., 2008	[91]
Lectin	120	Separation	Patterned electrode structures	25 µm	Deionized water, HEPES buffer	340 V_rms_	0.1 MHz (pDEP)	1.1 × 10^6^ V/m– 3.4 × 10^6^ V/m	Agastin et al., 2009	[78]
BSA	66	Separation	Patterned electrode structures	25 µm	Deionized water, HEPES buffer	340 V_rms_	0.1 MHz (pDEP)	1.1 × 10^6^ V/m– 3.4 × 10^6^ V/m	Agastin et al., 2009	[78]
Fibrinogen	340	Separation	Patterned electrode structures	25 µm	Deionized water, HEPES buffer	340 V_rms_	0.1 MHz (pDEP)	1.1 × 10^6^ V/m– 3.4 × 10^6^ V/m	Agastin et al., 2009	[78]
Prostate specific antigen (PSA)	34	Focusing	Parallel coplanar plate electrodes	7 µm	Phosphate buffer	0.1–0.5 V	23 Hz–1 kHz 47 Hz (optimum)	–	Gong 2010	[92]
Horseradish peroxidase	44	Trapping	Indium–tin-oxide (ITO) counter electrode, Tungsten cylinder nanoelectrode pins	2 µm	Ultrapure water, Dihydrorhodamine solution, Hydrogen peroxide	3.5 V	0.01 MHz	–	Laux et al., 2013	[93]
R-phycoerythrin (RPE)	240	Trapping	Cylindrical sub-microelectrodes	2 µm	Ultra-pure water	18 V_rms_	0.5 MHz (pDEP)	6 × 10^3^ V/m	Otto et al., 2014	[94]
IgG antibodies	150	Trapping	Cylindrical sub-microelectrodes	2 µm	Ultrapure water	14 V_rms_	0.1 MHz (pDEP)	6 × 10^3^ V/m	Otto et al., 2014	[94]
R-phycoerythrin	240	Trapping	Array of Ti nanogap electrode	5, 9 nm	PBS buffer	0.1–15 V_P-P_	0.1–4 MHz	3 × 10^17^ V^2^/m^3^	Lesser-Rojas et al., 2014	[95]
BSA	66.5	Trapping	Cylindrical nanoelectrodes array	2 µm	Ultrapure water	0.7–14.1 V_rms_	0.01 MHz (pDEP)	15 × 10^5^ V/m	Laux et al., 2015	[96]
BSA	66	Trapping	Gold nanocones, ITO electrode	1 µm, 500 nm, 250 nm, 125 nm	Aqueous solution of BSA	10 V_P-P_	2.5 MHz	–	Schäfer et al., 2015	[97]
Cardiac troponin I	26	Trapping	Gold concentration electrodes	25, 80 µm	TBE buffer	5 V_P-P_	0.001–1 MHz	–	Sharma et al., 2015	[98]
Enhanced green fluorescent protein	32.7	Trapping	Interdigitated electrodes	750, 450 nm	Ultrapure water	3.5 V_rms_	10–100 kHz	–	Laux et al., 2016	[99]
Amyloid β 42 protein Prostate specific antigen (PSA)	4.5 34	Trapping	Tantalum (Ta) and platinum (Pt) Interdigitated microelectrode	5 µm	PBS buffer	0.5 V_P-P_ 0.02 V_P-P_	50 MHz 50 MHz	–	Kim et al., 2016	[100]
Cardiac troponin I	30	Trapping	ITO coplanar electrodes	25 µm	PBS buffer	10 V_P-P_	10 kHz	–	Han et al., 2018	[101]
Amyloid β 42 protein	4.5	Trapping	Gold (Au) castellated microtip electrode array	40 µm	Deionized water	5 V_P-P_	60–100 MHz	5 × 10^4^ V/m	Al-Ahdal et al., 2019	[102]
Amyloid β 42 protein Tau-441	4.5 45.8	Trapping	Patterned interdigitated microelectrodes	3 µm	PBS buffer	0.5 V_P-P_ 0.6 V_P-P_	50 MHz	3.83 × 10^5^ V/m	Kim et al., 2019	[103]
**iDEP**										
Protein G Immunoglobulin G	150 21.8	Trapping	Nanopipette electrodeless DEP	∠3–6° ⌀100–150 nm	PBS buffer	−5, −1, +1, +2, +5 V	5 × 10^−7^ MHz	10^6^ V/m	Clarke et al., 2005	[50]
BSA	66.5	Trapping	Cylindrical insulating posts	520 µm	Deionized water NaOH K_2_HPO_4_	–	–	0.07 × 10^6^ V/m 0.09 × 10^6^ V/m (nDEP) 0.16 × 10^6^ V/m (nDEP)	Lapizco-Encina 2008	[49]
IgG	150	Focusing	Triangular and elliptical post	20 µm	Phosphate buffer	–	–	0.3 × 10^6^ V/m	Nakano et al., 2011	[75,77]
BSA	66.5	Focusing	Triangular and elliptical post	20 µm	Phosphate buffer	–	–	0.19 × 10^6^ V/m (pDEP)	Nakano et al., 2011	[75]
Amyloid-beta fibrils	-	Trapping	Insulating gradient sawtooth pattern electrodes	27 µm–10 nm	PBS buffer	400–1000 V (pDEP)	–	–	Staton et al., 2012	[89]
Streptavidin Anti-human IgG	52.8 150	Trapping and Separate	Insulating nanoconstrictions with nanoscale molecular dams	30 nm	PBS buffer NaCl	200–300 V_P-P_	0.01 MHz (pDEP) 1 MHz (nDEP)	150 V/m	Liao et al., 2012	[72,81]
BSA	66.5	Focusing	Nanoposts and nanopost arrays	–	Phosphate buffer	1500 V	–	–	Camacho-Alanis et al., 2013	[104]
Streptavidin	52.8	Trapping	Insulator constriction	0.1, 1, 10 µm	PBS buffer	–	100 kHz	30 × 10^3^ V/m	Chaurey et al., 2013	[68]
BSA	65	Trapping	Gold nanohole array, ITO electrode	⌀140 nm 600 nm	Water medium	6 V_P-P_	1 kHz	6 × 10^18^ V^2^/m^3^	Barik et al., 2014	[105]
Neuropeptide Y Orexin A	11 3.5	Trapping	Lateral constrictions, patterned glassy carbon electrode	50 nm	Phosphate buffer, PBS buffer	−0.7 V	3 MHz	300 V_P-P_/cm, 1.5 V/cm	Sanghavi et al., 2014	[106]
β-galactosidase, immunoglobulin G	465	Trapping	Triangular microposts	200 nm	Phosphate buffer, CHAPS	50–500 V	-	0.06 × 10^6^ V/m	Nakano et al., 2015	[30]
Strepavidin Phycoerythrin	52.8 240	Trapping	Nanoconstriction microchannel	150 nm	TBE buffer	200 V 470 V	60 Hz 20 Hz	2 × 10^8^ V/m	Chiou et al., 2015	[107]
Native Ribonuclease A (RNase A) Mono-PEG RNase A Di-PEG RNase A	13.7 33.7 53.7	Focusing	diamond-shaped insulating posts, platinum wire electrodes	10 µm	K_2_HPO_4_ buffer	500–4000 V	–	1 × 10^19^ V^2^/m^3^	Mata-Gómez et al., 2016	[108]
Prostate specific antigen (PSA) Anti-mouse immunoglobulin antibodies	30 150	Trapping	Lateral insulator constrictions nanoslit structure, glassy carbon modified platinum electrodes	50 nm	PBS buffer	–	4–6 MHz	70 V_rms_/cm 1.5 V_DC_/cm	Rohani et al., 2017	[109]
BSA	70	Trapping	Gold electrode constricted channel	1 µm	PBS buffer	0–2 V_DC_ 5–15 V_P-P_	0.01–100 kHz	–	Zhang and Liu, 2017	[110]
BSA Immunoglobulin G (IgG) Prostate-specific antigen (PSA)	66 150 34	Trapping	Ag/SiO_2_ Dense arrays of nanorods, sawtooth and castellated electrode arrays	5 µm	PBS buffer	5 V_P-P_	1–10 MHz 1 MHz 1 MHz	2.6 × 10^24^ V^2^/m^3^	Cao et al., 2018	[111]

## Abbreviations

DEP, dielectrophoresis; F_DEP_, dielectrophoresis force, AC, alternating current; DC, direct current; CM, Clausius–Mossotti; pDEP, positive dielectrophoresis; nDEP, negative dielectrophoresis; eDEP, electrode-based dielectrophoresis; iDEP, insulator-based dielectrophoresis; PDMS, polydimethylsiloxane; IgG, immunoglobulin G; BSA, bovine serum albumin; CHAPS, 3-[(3-cholamidopropyl) dimethylammonio]-1-propanesulfonate; HEPES, 4-(2-hydroxyethyl)-1-piperazineethanesulfonic acid; DNA, deoxyribonucleic acid; KCL, potassium chloride; PBS, phosphate buffered saline; RPE, R-phycoerythrin; BRB, Britton-Robinson buffer; and TBE, Tris-borate-EDTA.
